# Expression Profile of Hydroxysteroid Dehydrogenase-like 2 in Polychaete *Perinereis aibuhitensis* in Response to BPA

**DOI:** 10.3390/life13010010

**Published:** 2022-12-20

**Authors:** Yingpeng Li, Huan Zhao, Min Pang, Yi Huang, Boxu Zhang, Dazuo Yang, Yibing Zhou

**Affiliations:** 1 Key Laboratory of Marine Bio-Resources Restoration and Habitat Reparation in Liaoning Province, Dalian Ocean University, Dalian 116023, China; 2 Key Laboratory of Marine Eco-Environmental Science and Technology, First Institute of Oceanography, Ministry of Natural Resources of the People’s Republic of China, Qingdao 266061, China

**Keywords:** hydroxysteroid dehydrogenase-like 2, *Perinereis aibuhitensis*, BPA, gene and protein expression

## Abstract

Hydroxysteroid dehydrogenases (HSDs) play an important role in the metabolism of steroids and xenobiotics. However, the function of HSDs in invertebrates is unclear. In this study, we cloned the hydroxysteroid dehydrogenase-like 2 (HSDL2) gene in *Perinereis aibuhitensis*, which is 1652 bp in length, encoding 400 amino acids. This sequence contains conserved short-chain dehydrogenase and sterol carrier protein-2 domain, and the alignment analysis showed its close relationship with other invertebrate HSDL2. Further, the tissue distribution analysis of the *HSDL2* gene showed it is expressed strongly in the intestine. The expression level of HSDL2 after inducement with bisphenol A (BPA) was also detected both at transcriptional and translational levels. The results inferred that BPA exposure can induce HSDL2 expression, and the inductive effect was obvious in the high-concentration BPA group (100 μg/L). In summary, our results showed the detoxification function of HSDL2 in polychaetes.

## 1. Introduction

Bisphenol A (BPA), an important intermediate in the production of polycarbonate plastics, is a typical endocrine-disrupting chemical (EDC) [1]. It can be easily released into the environment through exposure to changes in temperature and pH and, hence, is widely distributed in the environment, with the concentration ranging from dozens of nanograms to hundreds of nanograms in water and sediment [2,3,4]. BPA has been shown to interfere with a variety of hormonal effects, and also induce immunotoxicity, mutagenicity, and carcinogenicity, and the adverse physiological effects of BPA have gained a lot of interest among researchers [5]. The adverse effects of BPA on reproduction and development are well documented not only in humans but also in many aquatic organisms [6]. Studies in zebrafish have demonstrated the endocrine disorders of BPA, such as infertility and precocious puberty [7,8]. In aquatic invertebrates, BPA has been reported to affect the embryonic development in sea urchin *Echinometra lucunter* L. and the molting in *Daphnia magna* Straus as well as in aquatic insects [9,10,11,12].

BPA can interact with estrogen receptors and act as a hormonal agonist due to its phenolic structure, which is similar to that of estrogen. It interferes with hydroxysteroid dehydrogenases (HSDs), which catalyze the interconversion between hydroxysteroids and oxysteroids [13]. HSDs belong to short-chain dehydrogenases (SDRs) or aldo-ketoreductases (AKRs), which play an important role in the metabolism of steroids, alcohols, fatty acid, prostaglandins, and xenobiotics [14]. There are many different types of HSDs, and each isoform varies in terms of tissue distribution, subcellular localization, cofactor preference, and pre-dominant catalytic direction [15,16]. Among these types, 3β-HSD and 17β-HSD are widely studied, and some research on the effect of BPA on 3β-HSD and 17β-HSD function in mammals and fishes has been carried out [7,17]. Ye et al. [17] found that BPA can influence the expression of HSD in mammals; 7.9 μM and 26.5 μM BPA, respectively, inhibited the expression of human testicular 3β-HSD and rat 3β-HSD. Mu et al. [7] reported that the HSD17b1 gene is significantly upregulated on exposure to BPA in zebrafish. However, it is unknown whether HSDs’ function in aquatic invertebrates is similar to that in vertebrates. With the development of genome sequencing, HSDs in mollusks have been studied, and some studies illuminated the reproductive function of HSDs such as HSD17b8 in *Chlamys farreri* (K. H. Jones & Preston, 1904) and HSD17b11 in *Haliotis diversicolor* Reeve [18,19]. However, the function of HSDs in other aquatic invertebrates such as polychaetes is still insufficiently understood.

Marine polychaetes are important invertebrates in the marine ecosystem with a wide geographical distribution, and they play an important role in aquatic food webs [20]. The characteristic of deposit feeding allows them to easily accumulate pollutants by ingestion or absorption through the body wall [21,22]. These characteristics have resulted in increasing use of polychaetes as biological indicators in ecotoxicological studies [20,23]. *Perinereis aibuhitensis* Grude is widely distributed along Asian coasts and estuaries. Its responses to heavy metal and organic pollutant exposure have been widely studied [24,25,26,27,28]. In our previous studies, we found that BPA exposure can promote the growth and development of oocytes in *P. aibuhitensis* and induce the expression of the G protein alpha subunit gene [29,30]. In order to further investigate the function of HSDs in *P. aibuhitensis* and the effect of BPA on HSDs, we cloned a novel *P. aibuhitensis* cDNA of SDR-hydroxysteroid dehydrogenase-like 2 (*HSDL2*) and expressed the recombinant protein in this study. Furthermore, the effect of BPA exposure on HSDL2 expression profiles was evaluated. The aim of this study was to identify the expression pattern of HSDL2 in response to BPA exposure and provide evidence for further investigation on the function of HSDs in *P. aibuhitensis*.

## 2. Materials and Methods

### 2.1. Polychaetes

*P. aibuhitensis* (1.5–2.5 g wet weight) was collected from Liaodong Bay in Panjing in China (121°35′ E, 40°45′ N), a relatively unpolluted site with a low ecological risk of BPA [31]. The animals were transferred to the laboratory and acclimated for a week before we initiated the experiment. Water was changed daily (temperature 17–18 °C, salinity 30–32, pH ±8.25). During acclimatization, the worms were fed with a commercial, formulated diet (13.0% moisture, 44.7% crude protein, 26.1% crude lipid, and 10.8% ash).

### 2.2. Cloning of HSDL2 Full-Length cDNA

#### 2.2.1. Extraction of Total RNA

A 0.1 mg amount of body wall of *P. aibuhitensis* was ground into a powder, and total RNA was extracted using TRIzol (TaKaRa, Dalian, China). The quality of RNA was examined using 1% agarose electrophoresis, the concentration of RNA was analyzed using an ultramicro spectrophotometer (529 ng/μL), and the ratio of OD260/OD280 was 1.91 (Thermal, CA, USA).

#### 2.2.2. Rapid Amplification of cDNA Ends of HSDL2 in *P. aibuhitensis*

A 1 μg amount of RNA was reverse transcribed into cDNA for the rapid amplification of cDNA ends (RACE) using a Clontech SMART RACE cDNA amplification kit (Clontech, CA, USA). The primers for *HSDL2* were designed using Primer Premier 5.0 software according to the partial sequence of *HSDL2* obtained from the transcriptome data which was constructed in our laboratory (BioProject accession number PRJNA841694) (Table 1).

The 3′- and 5′-RACE were conducted following the manufacturer’s protocols. The 3′-RACE reaction protocol was performed with a nested polymerase chain reaction (PCR). The outer PCR reaction conditions were as follows: denaturation at 94 °C for 1 min, followed by 30 cycles of denaturation at 94 °C for 10 s, annealing at 55 °C for 15 s, and extension at 68 °C for 1 min. Next, 1.0 μL of the outer product of 3′-RACE was used as the template for the inner PCR. The reaction conditions of the inner PCR were the same as that of the outer PCR. The 5′-RACE amplification was also conducted using nested PCR. The 5′-RACE reaction protocol was as follows: denaturation at 94 °C for 1 min, followed by 30 cycles of denaturation at 94 °C for 10 s, annealing at 56 °C for 30 s, and extension at 72 °C for 3 min. The outer PCR product of 5′-RACE was used as the template for the inner PCR. The reaction conditions of the inner PCR were also the same as that of the outer PCR. The PCR products were analyzed by electrophoresis on 1% agarose gel and then purified with an agarose gel DNA purification kit (TaKaRa, Dalian, China). The purified PCR products were sequenced by TaKaRa Biotechnology Co., Ltd. (Dalian, China).

#### 2.2.3. Bioinformatic Analysis of HSDL2

The similarity of this sequence to other known sequences was analyzed using the BLAST program (http://www.ncbi.nlm.nih.gov/BLAST/, accessed on 30 September 2021). The deduced amino acid sequence was obtained through the Expert Protein Analysis System (http://www.expasy.org/tools, accessed on 19 September 2021). The functional motif prediction in this sequence was performed using Motif Scan (http://www.hits.isbsib.ch/cgi-bin/PESCAN, accessed on 19 September 2021). The three-dimensional structure was analyzed using SWISS-MODEL (http://swissmodel.expasy.org/interactive, accessed on 27 May 2022). The sequence alignment was performed using ClustalW software (http://www.ebi.ac.uk/clustalW, accessed on 27 May 2022), and the phylogenetic analysis was conducted via Mega 5.0 software using the neighbor-joining algorithm. The tree topology was evaluated by 1000 replication bootstraps.

### 2.3. Tissue Distribution of HSDL2 Gene

The body wall, intestines, head, and parapodium of untreated polychaetes were sampled to detect the tissue-specific expression pattern of *HSDL2.* The specific primers in Table 1 were designed according to the full length of *HSDL2* cDNA. In accordance with previous studies, β-actin was chosen as the reference gene [29,32]. PCR efficiency was determined for each primer by constructing a standard curve using a series of diluted samples (Table 1). The PCR reaction was conducted in a 20 μL reaction mixture containing 10 μL of SYBR Premix Ex Taq II (TaKaRa, Dalian, China), 0.8 µL of each primer (10 mM), 0.4 μL of Rox Reference Dye (TaKaRa, Dalian, China), 2.0 μL of cDNA, and 6 μL of diluted water. The PCR was performed by the two-step method, and the reaction conditions were as follows: 40 cycles of 95 °C for 30 s, 95 °C for 5 s, and 60 °C for 34 s. The melting curve was analyzed after real-time quantitative analysis, and the PCR product was sent for sequencing to confirm accuracy.

### 2.4. Expression, Purification of Recombinant Protein, and Antibody Preparation

The conserved domain (1203 bp) of *HSDL2* in *P. aibuhitensis* was amplified by PCR. The PCR product was purified with a MiniBEST Agarose Gel DNA Extraction Kit (TakaRa, Dalian, China) and digested with *Bam*HI and *Sac*I to generate fragments with overhanging ends that could be ligated in the multiple cloning sites in the expression vector pET-28a (TaKaRa, Dalian, China). The recombinant plasmids were prepared and transformed into *Escherichia coli* (*E. coli)* BL 21 (DE3) cells (TransGen, Beijing, China), and the positive clones were selected by the plate coating separation method with 50 μg/mL kanamycin (Kan) (TransGen, Beijing, China) and 34 μg/mL chloramphenicol (Cm) (TransGen, Beijing, China) in Luria-Bertani (LB) solid medium (Sangon, Shanghai, China) and sent to TaKaRa Biotechnology Co., Ltd. for sequence analysis. The expression of HSDL2 was induced with 1 mM isopropyl-beta-D-thiogalactopyranoside (IPTG) (Solarbio, Beijing, China) at 37 °C for 4 h. The cells were suspended in phosphate-buffered saline (PBS) (TaKaRa, Dalian, China) and lysed by sonication on ice until they were no longer viscous and then centrifuged at 12,000× *g* rpm for 5 min. The protein expressed in the crude *E. coli* lysates, supernatant, and precipitation were detected by sodium dodecyl sulfate–polyacrylamide gel electrophoresis (SDS-PAGE), revealing that the protein expressed was mainly soluble protein. The protein in the supernatant was purified using Trap Talon crude (GE, Tokyo, Japan), and the concentration of purified protein (0.4 mg/mL) was measured with BCA Protein Assay Kits (Sangon, Shanghai, China).

The purified protein was sent to GenScript (Nanjing, China) to prepare antibodies with the concentration of 500 μg/mL, and the anti-HSDL2 rabbit serum was separated from New Zealand rabbits intraperitoneally injected with mixture of purified protein and Freund’s adjuvant. In order to detect the specificity of antibody, the protein was extracted from the body wall of untreated *P. aibuhitensis* and then transferred onto polyvinylidene fluoride (PVDF) membrane at 42 mA for 80 min. The membrane was blocked with 1.5% bovine serum albumin (BSA) in blocking buffer at 4 °C overnight. The membrane was probed with polyclonal anti-HSDL2 (1:2000) for 1 h at 37 °C. The membranes were washed three times for 5 min each time and AffiniPure Goat Anti-Rabbit IgG (H + L) (1:4000, Proteintech, IL, USA) conjugated to horseradish peroxidase (HRP) for 1 h at 37 °C. After three washes with TBST, the immunoreactive bands were visualized using diaminobezidin (DAB) substrate solution (OriGene, Wuxi, China).

### 2.5. The Expression Pattern of HSDL2 under BPA Exposure

#### 2.5.1. BPA Exposure

According to the preliminary experiment [30], three concentration groups of BPA (Sigma, Shanghai, China) (1, 10, and 100 μg/L) were set up for the experiment with three repetitions for each concentration. Meanwhile, a dimethyl sulfoxide (DMSO) (Sinopharm, Shanghai, China) solvent group (100 μL/L) and a seawater blank control group were set up. A total of 10 worms with similar weight were put into a 2 L glass beaker in each repetition and exposed to different concentrations of BPA. The worms were not fed during the experiment. The exposure experiment lasted 14 days with a water temperature of 18.0 ± 0.5 °C. The seawater in each treatment was renewed daily. The worms were sampled on day 4, 7, and 14, and the body wall of each sample was dissected and stored for quantitative PCR analysis.

#### 2.5.2. Real-Time Fluorescence Quantitative PCR for Gene Expression

Real-time PCR was used to detect the gene expression pattern under BPA exposure in *P. aibuhitensis*. The cDNA of samples in different treatments was synthesized following the instruction of PrimeScript^TM^ RT Reagent Kit With gDNA Eraser (TaKaRa, Dalian, China). β-actin was also used as the reference gene, and the specific primers for *HSDL2* and β-actin are shown in Table 1. The reaction condition was the same as that in Section 2.3.

#### 2.5.3. Indirect Enzyme-Linked Immunosorbent Assay

The protein expression of HSDL2 was detected by indirect enzyme-linked immunosorbent assay (ELISA). The protein was extracted using Western blotting and immunoprecipitation (IP) lysis buffer (Beyotime, Shanghai, China) from 0.2 mg body wall of *P. aibuhitensis*. The concentration of total protein was measured by a BCA Protein Assay Kit (Beyotime, Shanghai, China). Then, 100 μL of sample protein was added to the hole in a 96-well microtiter plate for coating overnight and blocked with 3% (*w*/*v*) BSA in phosphate-buffered saline (PBS) for 2 h at 37 °C. After incubation, the plates were washed with PBST three times for 5 min each. Further, 100 μL of anti-HSDL2 antibody (1:2000) was added to each well and incubated at 37 °C for 1 h. After incubation, the plates were washed with PBST three times for 5 min each. A 100 μL amount of HRP AffiniPure Goat Anti-Rabbit IgG (H + L) (1:4000) was added to each well. After incubation at 37 °C for 1 h, 100 μL of tetramethylbenzidine (TMB) substrate solution (Solarbio, Beijing, China) was added to each well. The plates were incubated at room temperature for 10 min in the dark. The reaction was stopped by adding 50 μL of ELISA terminator (Solarbio, Beijing, China). The absorbance values at 450 nm were measured.

### 2.6. Statistical Analysis

The relative gene expression of *HSDL2* was analyzed by the 2^−ΔΔCt^ method. All data are expressed as the mean ± standard error of mean (SEM). The gene and protein expression differences among BPA concentrations within sampling times were determined using one-way analysis of variance followed by Tukey’s test using SPSS19.0 software. A *p*-value < 0.05 indicated a statistically significant difference.

## 3. Results

### 3.1. Gene Sequence Analysis of P. aibuhitensis HSDL2

**Figure 1:**
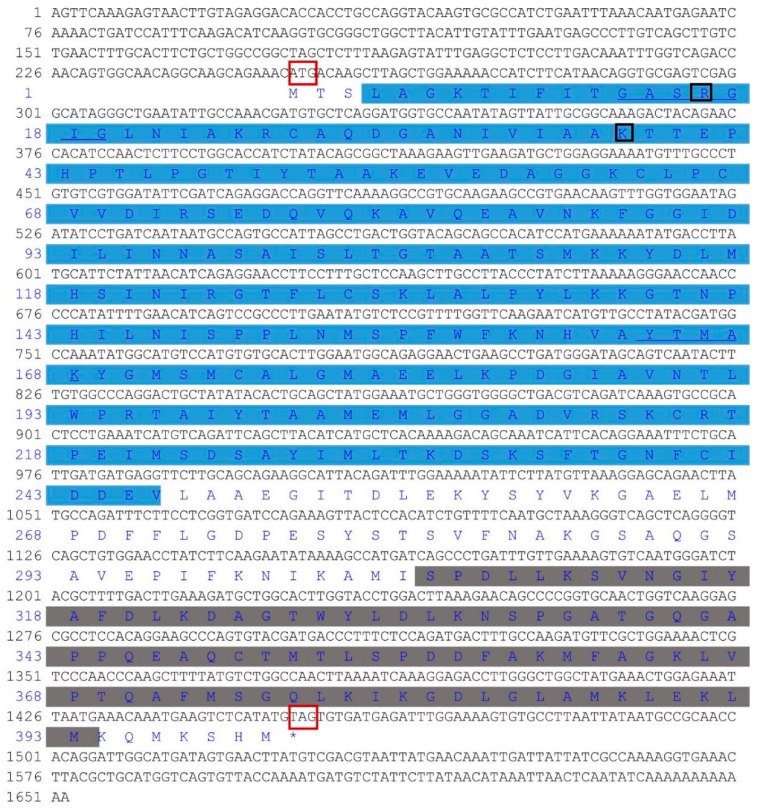
The deduced amino acid sequence of *P. aibuhitensis* HSDL2. The glycine-rich motif TGxxxGxG (12–19 aa) and the catalytic site YxxxK (164–168 aa) are underlined; the binding site for NADP(H) is highlighted with a black box. The initiation codon and termination codon are highlighted with a red box, * represents the end of codon. The SDR domain of HSDL2 is indicated by a shaded blue box (4–246 aa), and the SCP2-like domain is indicated with a gray box (306–393 aa).

**Figure 2:**
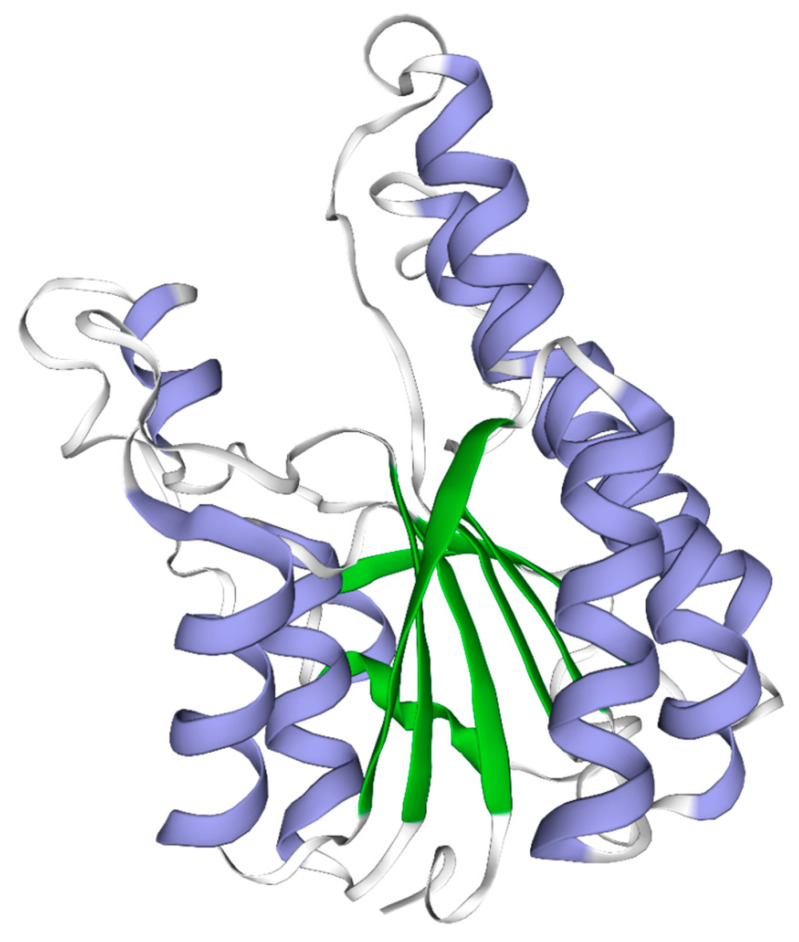
Prediction of the three-dimensional structure of the catalytic domain of *P. aibuhitensis* HSDL2. The beta strands are shown in green, helices are in blue, and the loop structure is shown in gray. The parallel β sheet is flanked by α helices.

The total length of *HSDL2* is 1652 bp, including a 251 bp 5′ untranslated region (UTR), 198 bp 3′ UTR, and 1203 bp open reading frame encoding 400 amino acids (Figure 1). The molecular weight of the deduced amino acid protein is 43,056 Da, with an estimated pI of 7.99. The sequence was submitted to the National Center for Biotechnology Information (NCBI) with GenBank number MH119076. This sequence consists of an N-terminal SDR domain of HSDL2 (4–246 aa), a C-terminal sterol carrier protein-2 (SCP2) domain (306–393 aa), the glycine-rich motif TGxxxGxG (TGASRGIG) (12–19 aa) necessary for cofactor binding, and the catalytic site YxxxK (YTMAK) (164–168 aa). Furthermore, arginine (R) at position 16 and lysine (K) at position 38 in this sequence indicate the binding for nicotinamide adenine dinucleotide phosphate NADP(H). The three-dimensional structure of the SDR domain displays a typical Rossmann fold with a central β sheet flanked by α helices (Figure 2) [15,33].

### 3.2. Comparison of the Peptide Sequence of HSDL2

**Figure 3:**
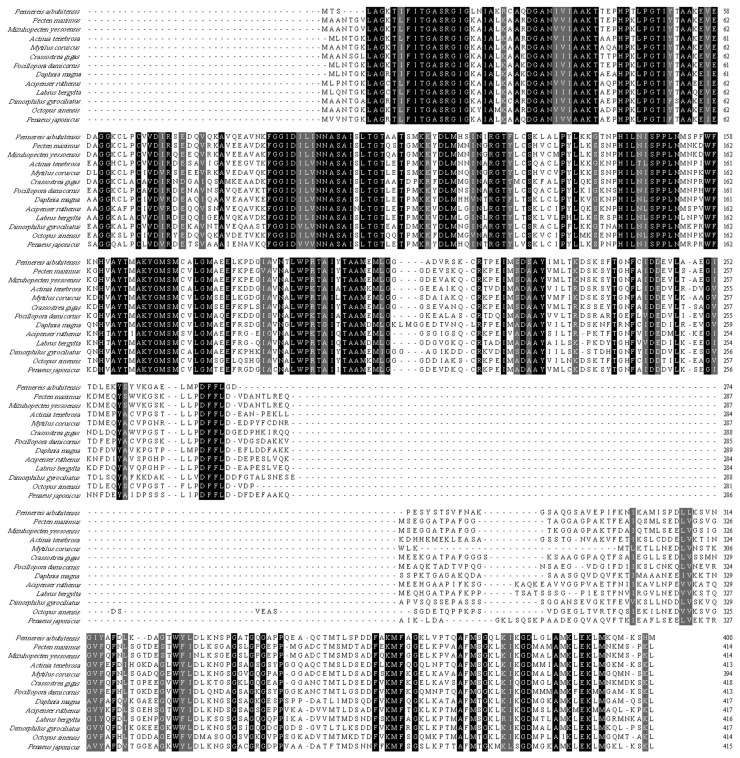
Alignment of HSDL2 protein with other known HSDL2 orthologues. Amino acid residues that are conserved in at least 50% of the sequence are shaded, and similar amino acids are shaded. The conserved motif (TGxxxGxG) and active site motif (YxxxK) are underlined. Accession numbers: *Pecten maximus* L. (XP_033738569.1), *Mizuhopecten yessoensis* Jay (XP_021379021.1), *Actinia tenebrosa* Farquhar (XP_031564327.1), *Mytilus coruscus* Gould (CAC5414625.1), *Crassostrea virginica* Gmelin (XP_022314551.1), *Pocillopora damicornis* L. (XP_027051106.1), *Daphnia magna* Straus (KZS05818.1), *Acipenser ruthenus* L. (XP_033891994.2), *Labrus bergylta* Ascanius (XP_020492596.1), *Octopus sinensis* d′Orbigny (XP_029649957.1), *Penaeus japonicus* Spence Bate (XP_042858890.1), *Dimorphilus gyrociliatus* O. Schmidt (CAD5114323.1).

The HSDL2 protein sequences of 12 invertebrate species were selected to be aligned with *P. aibuhitensis* HSDL2. This protein sequence shared the highest similarity with scallop *Pecten maximus* L. (67.7%), and it shared 59.8–67.0% sequence similarity with other invertebrates and fishes (Figure 3). The N-terminal SDR domain of HSDL2 in different species is highly conserved compared to the high diversity of C-terminal.

**Figure 4:**
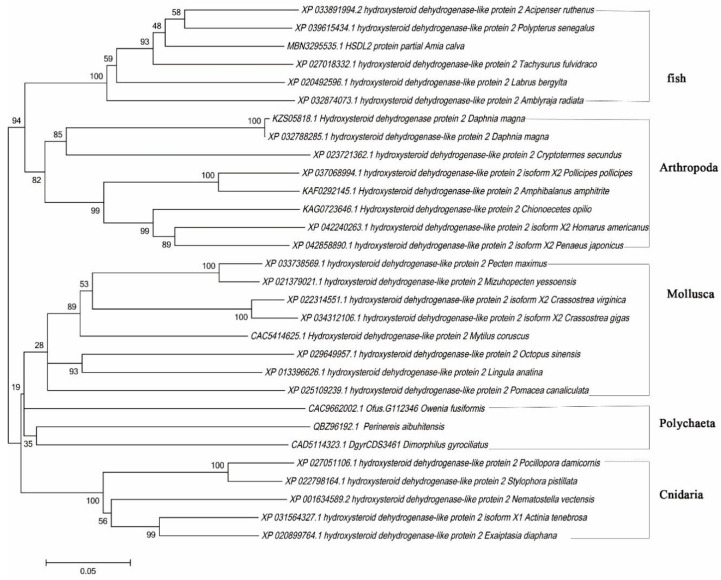
The phylogenetic tree of HSDL2 among various species. The values at the nodes are the percentages of 1000 bootstrap replicates.

Based on the alignment of the amino acid sequence, a phylogenetic tree was constructed. The result shows that the deduced amino acid sequence of *P. aibuhitensis* clustered mainly with invertebrates and was separated from vertebrates (Figure 4). The *P. aibuhitensis* HSDL2 was firstly grouped with polychaete *D. gyrociliatus* and *Owenia fusiformis* Delle Chiaje, and *P. aibuhitensis* HSDL2 was located closer to the Mollusca blade than to the Cnidaria blade. The HSDL2 homologs in Arthropoda were located closer to vertebrates than to other invertebrates.

### 3.3. Tissue Distribution of HSDL2

**Figure 5:**
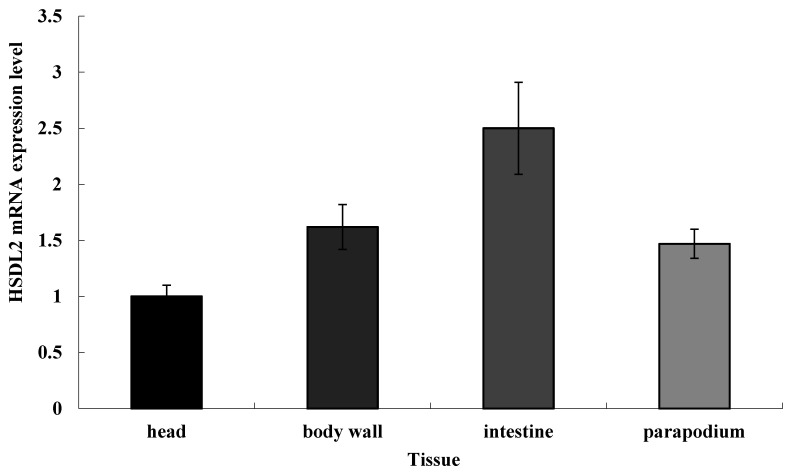
The HSDL2 mRNA expression pattern in different tissues of *P. aibuhitensis*. The relative expression level represented by mean ± standard error of mean (*n* = 4–6).

The gene expression of *HSDL2* in different tissues of *P. aibuhitensis* was determined. Figure 5 shows that *HSDL2* was expressed in all tissues with different levels. The expression level in the intestine was the highest, followed by that in the body wall and parapodium, and expression level in the head was the lowest.

### 3.4. Protein Purification of HSDL2 and Antibody Specificity

**Figure 6:**
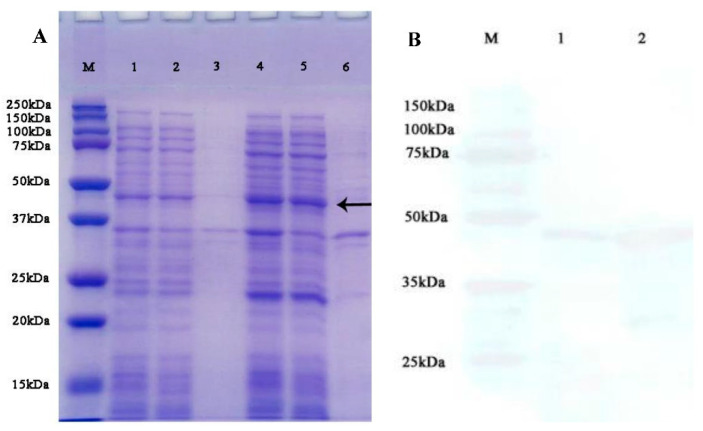
(**A**) SDS-PAGE; M-protein standards, 1—crude lysates of *E. coli* transfected with pET-28a, 2—lysate supernatant of *E. coli* transfected with pET-28a, 3—lysate pellet of *E. coli* transfected with pET-28a, 4—crude lysates of *E.coli* transfected with pET-28a-HSDL2, 5—lysate supernatant of *E. coli* with pET-28a-HSDL2, 6—lysate pellet of *E. coli* transfected with pET-28a-HSDL2; (**B**) Western blotting using anti-HSDL2 antibody; M—protein standards, 1 and 2—crude protein from untreated *P. aibuhitensis*.

The recombinant protein was expressed successfully as a His fusion protein with an expected molecular mass of 44 kDa, and it was expressed at a relatively higher level when the cells were incubated at 37 °C for 4 h with 1 mM IPTG. The results of SDS-PAGE showed a strong band in crude lysates and supernatant of recombinant protein (lane 4 and 5 in Figure 6A); it indicates that the majority of expressed protein existed in the supernatant. The specificity of the anti-HSDL2 antibody was detected using Western blotting, and only one brand was found (lane 1 and 2 in Figure 6B).

### 3.5. Gene Expression of HSDL2 under BPA Exposure

**Figure 7:**
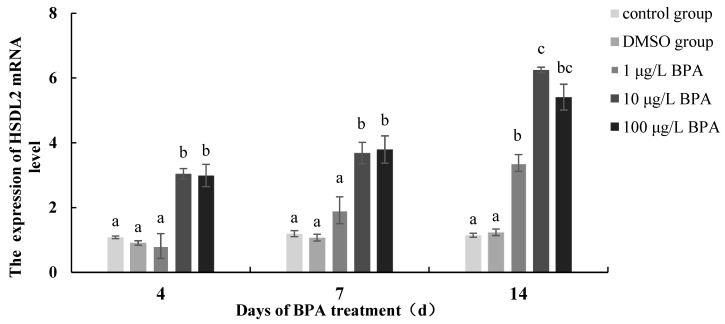
The *HSDL2* mRNA expression pattern under BPA treatment in *P. aibuhitensis*. The relative expression level represented by mean ± standard error of mean (*n* = 4–6), significant differences in different treatment at each sampling time was indicated by different letter (*p* < 0.05).

The gene expression of *HSDL2* under BPA exposure is shown in Figure 7. No significant difference was found between the blank control and solvent groups, indicating that the *HSDL2* gene expression change in this study was mainly influenced by BPA exposure. The gene expression of *HSDL2* in all concentration groups gradually increased with the exposure time. On day 4, the expression level of the *HSDL2* gene in the 1 μg/L BPA group was slightly lower than that in the blank control group (*p* > 0.05), while the mRNA expression level of *HSDL2* in the 10 and 100 μg/L BPA concentration groups was 2.81 and 2.76 times higher than that in the blank control group, respectively, with a significant difference (*p* < 0.05). On day 7, the mRNA expression level of *HSDL2* was significantly higher in the BPA group than in the control group except for the 1 μg/L group (*p* < 0.05), which was 1.60, 3.08, and 3.17 times, respectively, that in the control group. On day 14, the mRNA expression of *HSDL2* in each concentration group reached the maximum, being 2.95, 5.45, and 4.72 times higher than that in the control group, respectively (*p* < 0.05).

### 3.6. HSDL2 Protein Expression in P. aibuhitensis under BPA Exposure

**Figure 8:**
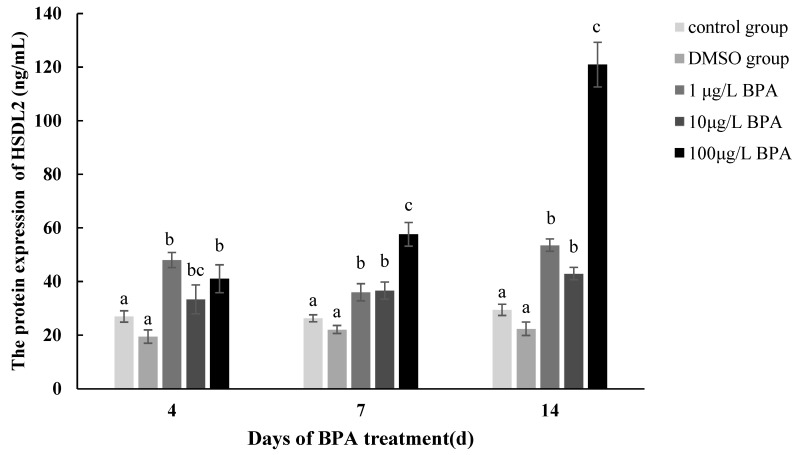
The HSDL2 protein expression pattern under BPA treatment in *P. aibuhitensis.* The protein expression level is represented by mean ± standard error of mean (*n* = 4–6); significant differences in different treatment at each sampling time are indicated by different letters (*p* < 0.05).

Figure 8 shows the protein expression changes in the HSDL2 induced by BPA. The results showed no significant difference between the blank control and DMSO solvent groups, which was similar to the gene expression results. The protein expression in the 1 μg/L BPA group firstly increased, then decreased, and finally increased, while the protein expression in other two groups, especially in the 100 μg/L BPA group, increased over time. On day 4, the expression in all concentration groups was higher than that in the control group (*p* < 0.05). The protein expression in the 1 μg/L, 10 μg/L, and 100 μg/L concentration groups was 48.04, 33.35, and 41.04 ng/mL, respectively. On day 7, the protein expression in the other two exposure groups, except the 1 μg/L group, was higher than that on day 4. The protein expression levels in the 1 μg/L, 10 μg/L, and 100 μg/L concentration groups were 36.00, 36.63, and 57.6 ng/mL, respectively (*p* < 0.05). On day 14, the protein expression in all exposure groups reached the maximum, with a value of 53.59, 42.99, and 120.96 ng/mL, respectively. The expression in the 100 μg/L group was significantly upregulated (*p* < 0.01).

## 4. Discussion

The SDR superfamily consists of a wide variety of NADP(H)-dependent oxidoreductases found in all three domains of life [15,33]. It is composed of 250–350 amino acids and is usually divided into two main types (classical and extended type) [34,35]. Although the variability in the structural and functional details are noted in this superfamily, with 15–30% sequence identity in pairwise comparisons, the most conserved feature in the N-terminal part is similar in each type. The N-terminal part contains a classical Rossman fold motif, which is critical for the structural integrity for SDRs, and this motif plays an important role in the binding of the nucleotide cofactor [34]. Furthermore, some other conserved motifs, such as the cofactor-binding motif and the active center motif, are also typical for SDRs [33,36]. The amino acid analysis and three-dimensional structure prediction in this study showed that this deduced amino acid sequence in *P. aibuhitensis* contains the conserved cofactor-binding motif TGASRGIG (12–19 aa) and YTMAK (164–168 aa). Additionally, a Rossman fold scaffold with a central β sheet flanked by α helices was noted, which indicates that this protein sequence belongs to the SDR subfamily (Figure 1 and Figure 2). The multiple sequence alignment and phylogenetic analysis showed that this protein sequence is closely related to the HSDL2 of other invertebrates and fishes. Therefore, we predicted that this sequence codes for HSDL2. A SCP2 domain in the C-terminal part was also observed in this sequence. The SCP2 domain, which is involved in binding sterols, has been found in SCP2 protein as well as the C-terminal of 17β-dehydrogenase. Burgardt et al. [37] analyzed the structure of SCP2 and found two or three subsets of residues (F, L, V, I, and M) in the structural core positions of SCP2. These highly frequent residues are of a bulky, hydrophobic character. In this sequence, the residues L and M were found in the SCP2 domain. The SCP2 domain of human HSDL2 contains a peroxisomal targeting signal (PTS) and is responsible for peroxisomal oxidation of fatty acids [38]. However, the peroxisome target sequence (ARL) was not found in this sequence, and the function of *P. aibuhitensis* HSDL2 in fatty acid metabolism needs further study.

*HSDL2*, a characterized SDR gene, was first identified in the human fetal brain cDNA library [39]. HSDL2 is highly expressed in the human liver, kidney, prostate, testes, and ovaries. However, little information is available about HSDL2 function in invertebrates compared with in humans. In this study, the gene expression of *HSDL2* was higher in the intestine than in other tissues. The relatively high expression in the intestine could be related to the contaminant accumulation via ingestion due to the characteristic of deposit feeding of *P. aibuhitensis*. In addition, the absorption via ingestion of *P. aibuhitensis* also absorbs xenobiotics via dermal contact, so the expression levels of *HSDL2* in the body wall and parapodium are relatively higher than in the head. In order to confirm the sufficient exposure to BPA, *P. aibuhitensis* was not feed during the exposure period, and dermal contact might be an important way to absorb xenobiotics. So, the body wall was selected as the target tissue in the further exposure study.

We expressed this gene in the bacterial expression system, and a soluble recombinant protein with expected molecular weight was obtained. Using purified HSDL2 as an antigen, a polyclonal antibody named anti-HSDL2 was prepared. As shown in Figure 6B, the anti-HSDL2 antibody reacted with crude protein from untreated worms, and only one band was observed, indicating the specificity of the polyclonal antibody.

BPA is relatively stable with a half-life of 38 days in water and 340 days in sediment [40], and Morales et al. [41] also reported that the nominal concentrations of BPA in the test medium remained constant during the exposure period in laboratory conditions. Based on these reports, we inferred that the concentrations of BPA in this experiment should be constant. In order to investigate the function of HSDL2 in *P. aibuhitensis*, the expression pattern under BPA exposure was measured both at transcriptional and translational levels. The protein expression showed a similar response to exposure concentration as gene expression, and both mRNA expression and protein contents were induced in a dose-dependent manner. It inferred that HSDL2 in *P. aibuhitensis* might play a role in the metabolism of BPA. Zhang et al. [42] reported the upregulation of the *HSDL2* gene in mantis shrimp *Oratosquilla oratoria* de Haan under lipopolysaccharide (LPS) challenge, which indicates a response to the LPS challenge. Our results showed a similar upregulation tendency as the results of Zhang et al. [42], indicating that invertebrate HSDL2 might positively respond to different substances. The gene expression of two kinds of 17β-HSD in *Mytilus galloprovincialis* Lamarck was downregulated in the digestive gland under BPA exposure both in female and male mussels, and the males seemed more susceptible to BPA exposure [43]. The change of 17β-HSD in mussels was different than the change in our results, and the difference in species and development stage might be one reason. Zhang et al. [43] used mature mussels for BPA treatments, and the worms used in our study were not in the breeding stage. Another reason might be related to the function of 17β-HSDs and HSDL2. It is noteworthy that 17β-HSDs display an important role in metabolism of sex steroids [13]. The function of HSDL2 in cholesterol metabolism and fatty acid metabolism has been inferred in mammals [33].

In *O. oratoria*, the author speculated that HSDL2 might function as a regulatory factor in the fatty acid metabolism, which is related to the SCP2 domain in the C-terminal part of HSDL2. Kowalik et al. [33] first analyzed the substrate specificity of human HSDL2; it showed no catalytic activity with several steroids and retinoids, but the peroxisomal localization of human HSDL2 suggests a possible involvement in fatty acid metabolism. Dai et al. [39] cloned an *HSDL2* gene in mice and found upregulating gene expression after inducement with cholesterol-containing food. Therefore, recent studies have paid more attention to the lipid metabolism of HSDL2 in various cancers [38,44,45,46]. However, all these studies focused on mammals, and the variability in gene structure among species makes functional differentiation possible. Some researchers reported the change of HSDL2 in the gonad maturation and sex hormone stimulation in aquatic organisms. The exposure of male rainbow trout *Oncorhynchus mykiss* Walbaum to 0.87 and 10 ng/L ethinylestradiol (EE_2_) induced *HSDL2* gene expression in a dose-dependent manner [47]. Xiao et al. [48] also found the existence of *HSDL2* in orange-spotted grouper (*Epinephelus coioides* Hamilton), and the expression of this gene was relatively high in the brain and gonad during sex reversal, which indicates the function of HSDL2 in the synthesis and metabolism of sex steroid hormones. Thongbuakaew et al. [49] identified *HSDL2* as a steroidogenesis-related gene in sea cucumber *Holothuria scabra* Jaeger. BPA is one kind of EDC. It is noteworthy that BPA can interact with estrogen receptors, and our previous study found that 10, 50, and 100 μg/L BPA could promote the growth and development of oocytes in immature *P. aibuhitensis* [30]. In this study the worms were not in the breeding stage, and BPA exposure induced the expression of HSDL2 in 10 and 100 μg/L BPA treatments both at a transcriptional and translational level. According to the reproductive toxicity of BPA on polychaetes, we supposed that HSDL2 might function as a steroidogenesis-related enzyme in the metabolism regulation of EDCs in *P. aibuhitensis*. Further study of the expression of HSDL2 during the development and reproduction stage of *P. aibuhitensis* should be conducted to verify this speculation.

It is well accepted that the metabolism of chemicals by organisms has an influence on bioaccumulation. The bioaccumulation of BPA in the tissues of fishes, mollusks, and other aquatic species has been detected [40]. It is reported that the BPA bioconcentration factors (BCFs) for fish range from 1.7 to 182, and the value is also low for mollusks, ranging from 4.5 to 144. The limited tendency to bioconcentrate in fishes might be due to the fast elimination of BPA by the metabolism. BPA can be rapidly converted to BPA glucuronic acid, as well as BPA sulfate, which is excreted in bile through the intestine in rainbow trout and zebrafish [40,50]. Species-specific difference in metabolism could cause the variety of BCF among species, and the characterization of the accumulation of BPA in *P. aibuhitensis* in further research could give more insights about the metabolism regulation of BPA.

## 5. Conclusions

In conclusion, the cDNA of *HSDL2* was cloned from marine polychaete *P. aibuhitensis*, and the functional motifs of HSDL2 were endowed with high conservation and an evolutionary relationship with their counterparts in other species. These characteristics indicate that this putative HSDL2 is a potential member of the HSD subfamily. This gene is expressed relatively high in the intestine of *P. aibuhitensis*. Transcript and protein levels of HSDL2 under BPA exposure were also analyzed to characterize its response to BPA exposure. The induction of HSDL2 by BPA exposure indicated that HSDL2 might be related to steroid biosynthesis in *P. aibuhitensis*. However, this work is preliminary, and the exact role of HSDL2 in *P. aibuhitensis*, as well as the connection between HSDL2 expression and the environment, needs to be further clarified.

## Figures and Tables

**Table 1:** The primer information in this experiment.

Primer Name	Sequence Information	Amplification Efficiency (%)	Sequence (5′–3′)
UPM (universal primer mix) long	RACE		CTAATACGACTCACTATAGGGCAAGCAGTGGTATCAACGCAGAGT
UPM short	RACE		CTAATACGACTCACTATAGGGC
HSDL2-F1	3′-RACE		CTAAAGGGTCAGCTCAGGGGTCA
HSDL2-F2	3′-RACE		GTGTCAATGGGATCTACGCTTTT
HSDL2-R1	5′-RACE		TTTTTCCAAATCTGTAATGCCTT
HSDL2-R2	5′-RACE		AATGATTTGCTGTCTTTTGTGAG
β-actin-F	Real-time PCR	99.96	GGGCTACTCCTTCACCACCA
β-actin-R	Real-time PCR		CGAAGTCCAGAGCAACATAG
HSDL2-F3	Real-time PCR	100.59	GAACATCAGTCCGCCCTTG
HSDL2-R3	Real-time PCR		GCTATCCCATCAGGCTTCAGTT
